# Infant Temperament, Breastfeeding, and Sleep at 6 and 14 Months

**DOI:** 10.3390/children13040559

**Published:** 2026-04-17

**Authors:** Nicki L. Aubuchon-Endsley, Ava R. Hanson, Emma Opoku, Shannon Snow

**Affiliations:** 1 Department of Psychology, Idaho State University, Pocatello, ID 83209, USA; 2 Department of Psychology, University of Tulsa, Tulsa, OK 74104, USA

**Keywords:** maternal psychophysiological stress, breastfeeding, infant temperament, infant sleep

## Abstract

**Highlights:**

**What are the main findings?**

- At 6 months, greater maternal prenatal salivary cortisol concentration was related to poorer infant sleep quantity, greater breastfeeding frequency was associated with decreased infant sleep quality, and greater infant Surgency was associated with better infant sleep quality at 14 months.
- None of the interactions between infant temperament and breastfeeding frequency were statistically significant. However, conditional effects highlight patterns of relations between infant temperament and breastfeeding frequency that future studies should investigate in relation to infant sleep quality.

**What are the implications of the main findings?**

- The findings support the transactional model of sleep development by highlighting maternal–infant factors related to infant sleep quantity or quality at 6 or 14 months of age including in utero exposure to stress hormones, infant temperament, and breastfeeding frequency.
- Future studies should investigate reciprocal, dynamic interactions among these factors in a more comprehensive fashion, including preliminary directions of effects highlighted via the current study results.

**Abstract:**

**Background/Objectives:** Sufficient sleep quantity/quality in infancy is crucial for healthy development, so it is important to identify early associated predictive factors. Research findings highlight salient endogenous (infant temperament) and exogenous (breastfeeding) influences, though no known studies have examined nuanced and interactive relations among these variables from early to late infancy/toddlerhood. Thus, the current study examined the main and interaction effects of these variables on infant sleep at 6 and 14 months while controlling for prenatal cortisol exposure. **Methods:** Data from a subsample (*n* = 79) of the Infant Development and Healthy Outcomes in Mothers Study were used, including prenatal maternal saliva samples assayed for cortisol and maternal questionnaires that included retrospective reporting of infant temperament, sleep quality and quantity, and breastfeeding frequency. **Results:** Multiple linear regression results include a statistically significant negative relation between prenatal maternal cortisol area under the curve and 6-month infant sleep quantity. A greater breastfeeding frequency at 6 months was associated with decreased 6-month sleep quality via conditional but not interaction effects. Greater 6-month infant Surgency was associated with better sleep quality at 14 months. There were no statistically significant interaction effects. **Conclusions:** The findings suggest that maternal psychophysiological stress has a significant influence on infant sleep duration, while research should further investigate the role of infant temperament and breastfeeding in shaping infant sleep quality. Significant conditional effects highlight patterns that should be re-examined with a larger sample to determine whether infant temperament may buffer against negative associations between breastfeeding frequency and infant sleep quality in early and late infancy in a developmental stage-consistent manner. Future replication studies should include a multi-method, longitudinal assessment of all key study variables, as well as a larger, more sociodemographically diverse sample of maternal–infant dyads.

## 1. Introduction

Infant sleep quantity and quality are foundational to infant health and development as well as familial bonding and wellbeing [1]. Though infant sleep problems are common, there are gaps in the literature regarding predictors of long-term sleep quantity and quality utilizing longitudinal methods, as well as the consideration of associated maternal–infant moderating and mediating factors. Preliminary evidence suggests that maternal breastfeeding and infant temperament are concurrently important when examining infant sleep quality and quantity [2]. It is also crucial to consider underlying biological contributors like maternal cortisol concentrations that may influence the associations between breastfeeding and sleep. In addition, these multivariate relations may change over time due to development across infancy/toddlerhood and the resulting maternal–infant interactions. Therefore, in the current study, researchers examined whether maternal breastfeeding moderates the relation between infant temperament and infant sleep quality and quantity at 6 and 14 months of age while considering maternal prenatal diurnal cortisol concentrations.

### 1.1. Infant Sleep

Researchers suggest that infants’ need for sleep varies with age, with 14–17 h, 12–15 h, and 11–14 h being recommended at birth to 3 months of age, 1 to 11 months of age, and 1 to 2 years of age, respectively [3]. Infant sleep difficulties are a common concern among parents, such that an estimated 17–27% of infants experience sleep problems [4,5]. Common sleep problems among infants include frequent late-night awakenings, sleep fragmentation or repeated sleep interruptions [6], and infant sleep deprivation [7].

Sleep is essential for physical restoration, learning, memory, and neurocognitive and neurobehavioral performance [8] and is especially important during infancy, which represents a sensitive time in development. In particular, a review by Field [9] found that infant sleep problems are related to a broad range of short- and long-term negative outcomes. This includes altered growth trajectories and an increased risk for obesity, sudden infant death syndrome (SIDS), and obstructive sleep apnea [7,10]. Additionally, mental health outcomes include poor socioemotional development, internalizing and externalizing symptoms, deficits in neurodevelopment, and associated cognitive skills like attention regulation [11,12,13]. Given the prevalence and implications of infant sleep difficulties, researchers have attempted to identify risk and protective factors associated with sleep difficulties, which may inform prevention and intervention strategies. Both infant and parental factors have emerged as significant predictors of sleep outcomes. Specifically, infant temperament has been found to influence infant overall sleep quality.

### 1.2. Infant Temperament and Sleep

Studies often approach temperament through the context of difficult and easy infant temperament, with 20% of infants being defined as having a difficult temperament by De Marcas et al. [14], including irritability, excessive crying, and fussy behavior. Gartstein et al. [15] and Putnam et al. [16] identify three facets of infant temperament: Surgency/Extraversion (approach-oriented behavior), Negative Emotionality (fearful or angry behavior), and Orienting/Regulation (control of emotional reactivity). Like both Gartstein et al. [15] and De Marcas et al. [14], Spruyt and colleagues [17] define difficult temperament in terms of difficulty adapting, withdrawn behavior, and negative mood. Infant factors related to better sleep outcomes include temperament facets like greater Surgency/Extraversion including Vocal Reactivity [15]; less Negative Emotionality including less Distress to Limitations and Sadness [15]; and greater effortful control/Regulation including less negative reactivity [18], hypersensitivity, and hyposensitivity [14], shorter duration of Orientation, and greater Soothability [15].

These key factors are related to several types of infant sleep outcomes. For instance, infants with difficult temperament experience increased sleep concerns compared to those with an easy temperament, including difficulties initiating sleep and increased sleep fragmentation [15]. Additionally, infants with easy temperament sleep more than those with difficult temperament [17]. Similarly, infants’ negative mood is related to sleep fragmentation and prolonged night awakenings [19]. Furthermore, infants with negative mood have greater difficulty initiating and maintaining sleep [20]. Relations between infant temperament and sleep may be bidirectional. For example, researchers have found that delayed bedtime and decreased sleep duration result in worse temperament in terms of increased anxiety and decreased inhibition [9].

### 1.3. Breastfeeding and Infant Sleep

Relations between infant temperament and sleep should also be considered within the context of salient parental factors [9], such as maternal physical and emotional availability [21], breastfeeding [22], paternal caregiving [23], and maternal mental health (e.g., perinatal depression and anxiety symptoms) [24]. The particularly robust relation between breastfeeding and sleep is well documented within the existing empirical literature and may reflect underlying biopsychosocial mechanisms.

One possible mechanism may be that breastfeeding mothers demonstrate an “increased sensitivity” [25] (p. 19) toward their infant’s crying. Breastmilk release is facilitated by the secretion of oxytocin from the hypothalamus [26,27], which is a hormone associated with “social competence and adequate caregiving” [27] (p. 2) and decreased feelings of stress [26]. Oxytocin facilitates an emotional connection between parents and their infants [28], which may explain why there are reports of increased night awakenings among breastfed infants. For instance, research indicates that breastfed infants experience reduced sleep consolidation and prolonged night awakenings compared to formula-fed infants [6,29,30]. However, breastfed infants are more likely to sleep for a longer duration at night than formula-fed infants, which extends into early childhood [21]. As an example, Jafar et al. [21] concluded that infants who are breastfed at 6, 9, 12, and 24 months of age achieve greater sleep duration than formula-fed infants. Notably, formula-fed infants, partially breastfed infants, and fully breastfed infants slept an average of 11.22 h, 12.12 h, and 12.86 h per night, respectively [21]. Likewise, Ramamurthy et al. [30] discovered that breastfed 6-month-old infants show an increased sleep duration compared to formula-fed infants.

These findings imply that maternal breastfeeding benefits overall sleep duration, with additional factors contributing to the relations between breastfeeding and sleep quality (e.g., breastfeeding patterns across age). Therefore, it may be helpful to concurrently consider parental and infant risk/resiliency factors to better understand these complex relations. In particular, preliminary research has examined interactions between infant temperament and breastfeeding in predicting infant sleep outcomes.

### 1.4. Interactions Between Breastfeeding and Infant Temperament in Predicting Sleep

Although Sasaki et al. [31] did not find a significant interaction between infant temperament and breastfeeding in relation to infant sleep, they found that more frequent breastfeeding and increased Surgency and regulatory behaviors at 6 months were associated with decreased sleep quality at 14 months. Similarly, Cohen-Engler et al. [29] found breastfeeding to be associated with increased sleep duration as well as fewer episodes of infant crying and irritability. Weinraub et al. [32] found that increased awakenings occurred most often among breastfed infants and those with difficult temperament at 6 to 15 months of age. Thus, breastfeeding and temperament both influence infant sleep quantity and quality, though breastfeeding may have different associations between infant sleep quantity and infant sleep quality. Furthermore, breastfeeding and temperament may also be bidirectionally related.

Infants with positive reactions to breastfeeding (e.g., pleasant affectivity, laughing, smiling) may be breastfed more often to elicit desired behaviors [15]. Gartstein et al. [15] discovered that surgent infants have a greater caloric need and require more breastfeeding. Findings by Krol and Grossman [33] add that breastfeeding benefits the socioemotional development of infants through findings of increased prosocial behavior and decreased aggressive behaviors among infants due to the release of oxytocin through breastmilk. In contrast, Glynn et al. [34] propose that breastfeeding may negatively influence temperament, specifically when significant levels of the stress hormone cortisol are present in breastmilk. A high concentration of maternal cortisol in breastmilk corresponds with increased fearful behavior among infants [34]. Therefore, important biomarkers like maternal cortisol should be considered when examining interactions between maternal breastfeeding and infant temperament.

### 1.5. Current Study Justification and Aims

Taken together, these findings suggest that easier infant temperament may positively influence sleep quality and quantity in the long-term, and the effects of infant temperament on infant sleep quality may be moderated by breastfeeding. However, the influence of breastfeeding on infant sleep may decrease after 6 months, possibly due to the decreased frequency of breastfeeding at this time. Additionally, there is a bidirectional relation between infant temperament and breastfeeding such that better temperament leads to more breastfeeding and more breastfeeding leads to more positive infant affect and less crying and irritability. Therefore, breastfeeding and temperament may interact to predict infant sleep duration and quality, though the latter may show a different direction of effects in early (negative associations) versus late (positive associations) infancy as nightly breastfeedings become less frequent with the introduction of complementary and solid foods. Moreover, associations between breastfeeding and infant temperament may depend on cortisol in breastmilk, so controlling for prenatal cortisol exposure when examining relations among infant temperament, breastfeeding, and infant sleep is an important consideration [31]. However, more studies are needed to explore these nuanced and interactive relations over the course of infancy given that no known longitudinal study has integrated these factors into a unitary multivariate model and tested the model from early to late infancy/toddlerhood. Thus, the current study proposes to examine these models, evaluating the main and interaction effects of infant temperament and breastfeeding on infant sleep duration and quality at 6 and 14 months of age.

### 1.6. Hypotheses

It is hypothesized that (1) there will be significant main and interaction effects of easier infant temperament (less Negative Emotionality and greater Surgency and Regulation at 6 months) and greater breastfeeding frequency on greater infant sleep quantity at 6 and 14 months of age while controlling for indicators of in utero cortisol exposure (cortisol awakening response (CAR) and area under the curve with respect to ground (AUCg)) that are associated with outcome variables. It is further hypothesized that (2) there will be significant main effects of easier infant temperament (less Negative Emotionality and greater Surgency and Regulation at 6 months) and less frequent breastfeeding on greater infant sleep quality at 6 months of age while controlling for indicators of in utero cortisol exposure (CAR, AUCg) that are associated with outcome variables. The two primary predictors will also interact such that easier infant temperament (less Negative Emotionality and greater Surgency and Regulation at 6 months) will serve as a buffer to relations between greater breastfeeding frequency and poorer infant sleep quality at 6 months of age. However, these main and interaction effects are hypothesized to shift in direction at 14 months of age. In other words, it is hypothesized that (3) there will be significant main and interaction effects of easier infant temperament and greater breastfeeding frequency on greater infant sleep quality at 14 months of age while controlling for indicators of in utero cortisol exposure (CAR, AUCg) that are associated with outcome variables.

## 2. Materials and Methods

### 2.1. Procedure

The present study will test hypotheses using pre-existing data collected within the Infant Development and Healthy Outcomes in Mothers (IDAHO Mom) Study. The IDAHO Mom Study was a longitudinal research project that encompassed a variety of variables related to maternal health and infant development [35,36]. The procedure for the larger study consisted of a prenatal session at 33–37 weeks gestation and follow-up sessions, including at 6 months and 13–14 months postpartum (±2 weeks). During the prenatal session, trained research assistants administered interviews and self-report questionnaires and collected demographic information while providing instructions and support with written informed consent procedures. Following the prenatal session, participants were given a saliva collection kit and instructions to measure diurnal cortisol for 3 days. At the 6-month postnatal session, participants completed clinical interviews and additional self-report measures. The present study, which includes the principal investigator (PI) of the IDAHO Mom Study, Dr. Nicki Aubuchon-Endsley, in addition to research assistants at the University of Tulsa, focuses on responses to the Infant Behavior Questionnaire Revised: Short Form (IBQ-R SF) at 6 months, Infant Dietary and Breastfeeding Questionnaire at 6 and 14 months postpartum, and the Infant Health and Sleep Questionnaire at 6 and 14 months postpartum, as well as prenatal diurnal cortisol concentrations.

#### Recruitment

IDAHO Mom Study participants were recruited from medical centers, local businesses, schools, libraries, community centers, the local tribal nation, and childcare facilities in a health professional shortage area (HPSA) in a Northwestern region of the U.S. Participants were recruited via print and online advertisements (e.g., flyers, social media) and phone-screened for eligibility. If participants were adolescent mothers (<18 years old), mothers of advanced maternal age (>35 years old) or had multiple pregnancy/births, health conditions impacting endocrine functioning (e.g., gestational diabetes, pre-eclampsia, HIV, and toxemia), mental health diagnoses/symptoms (e.g., schizophrenia or bipolar disorder), chronic use of recreational substances (e.g., marijuana, or cocaine), use of Food and Drug Administration (FDA) category D or X medications, or use of alcohol (>40 drinks) during pregnancy, then they were deemed ineligible for study participation [35]. Participants were compensated for their participation with a monetary award of $30 per session and $5 for each day of completed saliva collection for a total of $165 if all study sessions/components were completed. The study was conducted from 2015 to 2018 and was approved by Idaho State University’s Human Subjects Committee (protocol 4191). Please see Riedstra and Aubuchon-Endsley [35] for a CONSORT diagram of recruitment, enrollment, and retention of participants over the course of the longitudinal study.

### 2.2. Measures

#### 2.2.1. Infant Temperament

The Infant Behavior Questionnaire Revised: Short Form (IBQ-R SF) uses 91 parent-report items to assess temperament according to 3 scales and 14 subscales. The IBQ-R SF defines temperament as individual differences in reactivity and self-regulation influenced over time by heredity and experience [37]. The IBQ was developed by Mary Rothbart in 1981 with six scales [38]. The measure was revised (IBQ-R) to include eight new scales to reflect new understandings of temperament [39]. In this study, the participant reported the frequency with which the infant displayed specific behaviors during the past 2 weeks on a 7-point scale from “never” to “always.” Putnam et al. [16] developed a short form of the IBQ-R to abbreviate the scales but still approximate the full content of the original measure. Longitudinal stability and test–retest reliability of the short form scales were similar to the standard form with a test–retest reliability average of 0.72, ranging from 0.54 to 0.93 [16]. The items are categorized based on the scale they reflect. The scales include Surgency, Regulation/Orientation, and Negative Affectivity while the subscales include Approach, Vocal Reactivity, High-Intensity Pleasure, Smiling and Laughter, Activity Level, Perceptual Sensitivity, Sadness, Distress to Limitations, Fear, Rate of Recovery from Distress, Low-Intensity Pleasure, Cuddliness, Duration of Orienting, and Soothability.

In this study, scales within the IBQ-R SF were operationalized within constructs of Surgency, Regulation/Orientation, and Negative Affectivity. The subscales referenced as Surgency were Approach, Vocal Reactivity, Smiling and Laughter, Activity Level, Perceptual Sensitivity, and High-Intensity Pleasure. The subscales defined as Regulation/Orientation included Duration of Orienting, Cuddliness, Soothability, and Low-Intensity Pleasure. The subscales of Sadness, Distress to Limitations, Fear, and Rate of Recovery were considered Negative Affectivity. The IBQ-R has been assessed for reliability in other studies involving infants between 3 and 12 months of age. Longitudinal confirmatory factor analyses revealed a model fit for the IBQ-R from 2 weeks to 12 months with 0.69 for Surgency, 0.78 for Negative Affectivity, and 0.73 for Regulation/Orientation [40]. Reliability analyses in the current study revealed the internal consistency of Cronbach’s *a =* 0.79, 0.56, and 0.66 for Surgency, Negative Affectivity, and Regulation/Orientation, respectively.

#### 2.2.2. Breastfeeding Frequency

The Infant Dietary Questionnaire (IDQ) measures maternal breastfeeding behaviors since birth such as formula feeding, duration and frequency of breastfeeding, consumption of solid food, supplement use, and difficulties breastfeeding. It is a 7-item self-report questionnaire adapted from the Pregnancy Risk Assessment Monitoring System (PRAMS). The PRAMS samples a diverse group across the U.S. The questions have been perfected across the eight versions from 1988 to 2021 [41]. For this reason, the IDQ referenced the PRAMS for appropriate formatting of inquiries related to breastfeeding behaviors. This study used breastfeeding frequency to measure this construct, which was assessed by the following questions, including “Are you currently breastfeeding your infant/toddler?” and “Presently, for how many feedings a day do you breastfeed?” if participants indicated current breastfeeding. The participants who indicated that they are not currently breastfeeding had values of “0” for the breastfeeding frequency variable at each associated session.

#### 2.2.3. Infant Sleep Quality and Quantity

The Infant Health and Sleep Questionnaire (IHSQ) was adapted from the Infant Feeding Practices Study II (IFPS II), a longitudinal perinatal questionnaire developed by the FDA and Center for Disease Control and Prevention (CDC) [42], as well as the Brief Infant Sleep Questionnaire (BISQ) [43]. The resulting IHSQ is a 29-item maternal-report questionnaire. Items 1–9 ask about previous illnesses, medications, immunizations, and hospitalizations of the infant. Items 10–17 ask about sleep quality on a Likert scale. Participants rate phrases such as “My baby sleeps through the night” and “My baby has a regular bedtime” on a scale of 1 (not like your baby) to 5 (very much like your baby). In item 18, the participant reports the infant’s usual bedtime.

Infant sleep quality and quantity reported at the 6- and 14-month sessions were used for this study. For scoring infant sleep, items 10–13 and 15–17 were quantified and averaged to reflect sleep quality. Reliability analyses revealed that item 14 showed minimal correlation with the other items in the scale at each time period, so this item was removed. Moreover, the internal consistency of the overall scale was improved at 6 (Cronbach’s *a =* 0.83) and 14 (Cronbach’s *a =* 0.80) months with removal of that item. For measuring sleep quantity, item 20, which asked “How many hours of sleep did your infant/toddler get last night?,” was quantified.

#### 2.2.4. Salivary Cortisol Concentrations

Participants were asked to provide saliva samples for 3 days to assess the cortisol awakening response (CAR) and the area under the curve with respect to ground (AUCg). CAR serves as an index of early-morning cortisol level peak, which has been linked to greater psychophysiological stress, prenatal depression symptom severity, and associated health outcomes in perinatal women [44,45]. AUCg is an index of diurnal cortisol output and has also been linked to peripartum maternal mental health symptoms [46]. CAR and AUCg are biomarkers of hypothalamic–pituitary–adrenal (HPA) axis or neuroendocrinological functioning, and maternal prenatal values have been linked to important infant outcomes, including growth [47]. Examining cortisol in these ways allows for a more comprehensive examination of the constructs of interest in the present study.

In order to quantify cortisol concentration values, participants were asked to provide passive drool saliva samples immediately upon waking up in the morning, 30 and 45 min after waking up, and right before bed [47]. Saliva collection times were confirmed via participant logs and responses to automatic text messages, when available. Standardized instructions were used to minimize potential contaminants (e.g., no eating, smoking, or brushing teeth prior to providing the sample) and maintain the integrity of the samples (e.g., storage of sample in participants’ and then lab freezers). Duplicate Salimetrics (Carlsbad, CA, USA) enzyme-linked immunosorbent assay (ELISA) kits (sensitivity < 0.007 μg/dL, assay range = 0.012–3.00 μg/dL) were used to quantify cortisol concentrations at each time point. Implausible individual values (e.g., >4 ug/dL) were removed. The difference between the first two timepoints allowed for the quantification of CAR, while AUCg was calculated using an established trapezoid formula [48] used extensively in perinatal populations. CAR and AUCg were calculated for each day, and a mean of the 3 days was computed for the current study.

### 2.3. Statistical Analyses

Least squares linear multiple regression analyses using the PROCESS macro [49] were used to examine the proposed relations between each scale of infant temperament (i.e., Regulation/Orientation, Surgency, Negative Affectivity), the moderator variable of breastfeeding frequency, and each criterion variable (i.e., sleep quantity and quality) at each postnatal age (i.e., 6 and 14 months) for a total of 12 models. Univariate normality was primarily assessed by examining values of skewness and kurtosis, but tests of normality (e.g., Shapiro–Wilk) were also considered. The variable of sleep quantity at 6 months was winsorized to correct for outliers. Sleep quality at 6 months and the covariate AUCg received a cube root and log base 10 transformation, respectively, to correct for the violation of the assumption of normal distribution of data for each variable. Visual inspection of frequency histograms revealed a normality of distribution for the respective corrected and uncorrected variables. The remaining assumptions were not violated for all variables, as evidenced by the examination of scatterplots to ensure an absence of range restriction and residual plots to test for the normality of residuals and homoscedasticity [49]. The data were analyzed using SPSS 28.0.1.1 (IBM, Armonk, NY, USA).

## 3. Results

### 3.1. Sample Characteristics

Sociodemographic descriptive characteristics can be seen for mothers with complete data at 6 (*n* = 79; *M_AGE_* = 26.97 years, *SD_AGE_* = 4.04 years) and 14 (*n* = 44) months postpartum (see Table 1). Most were married and identified as White regarding their ethnoracial identity (see Table 1). The sample heavily comprised individuals with a reported income of at least $50,000/year. Almost all participants in the sample possessed a high school degree, with the majority having at least a college degree. This sample’s sociodemographic composition closely aligns with 2024 Idaho Census data [50].

### 3.2. Descriptive Statistics

For the facets of infant temperament at 6 months, average scores on the Regulation/Orientation scale were the highest (*M* = 5.19, *SD* = 0.47) followed by scores on the scale of Surgency (*M* = 4.95, *SD* = 0.55), with average scores being the lowest on the scale for Negative Affectivity (*M* = 3.08, *SD* = 0.63). Mothers reported their average daily breastfeeding frequency at 6 and 14 months to be 3.71 (*SD* = 3.98) and 1.33 (*SD* = 2.28), respectively. The average sleep quality at 6 months was 3.75 (*SD* = 0.83) on a five-point Likert scale. The sleep quality slightly increased at 14 months, with an average reported score of 4.14 (*SD* = 0.68). The average daily sleep quantity steadily increased from the 6-month postnatal value of 9.53 h (*SD* = 1.94 h) to the 14-month follow-up session value of 10.58 h (*SD* = 1.23 h; See Table 2).

### 3.3. Covariates

It was hypothesized that controlling for prenatal cortisol exposure when examining relations among infant temperament, breastfeeding, and infant sleep may be necessary due to research findings that associations between breastfeeding and infant temperament may depend on cortisol in breastmilk [31,34,51]. Therefore, prior to running regression analyses, bivariate correlation analyses were completed to examine the association between the proposed covariates of CAR and AUCg, and the outcome variables of sleep quantity and quality at 6 and 14 months. Contrary to some preliminary findings, CAR was not correlated with any of the outcome variables, while AUCg was only statistically significantly correlated with sleep quantity at 6 months (*r* = −0.35, *p* = 0.007). Therefore, AUCg was only included as an individual covariate for analyses including sleep quantity at 6 months.

### 3.4. Primary Analyses

#### 3.4.1. Hypothesis 1

There will be significant main and interaction effects of easier infant temperament and greater breastfeeding frequency on greater infant sleep quantity at 6 and 14 months of age while controlling for indicators of in utero cortisol exposure that are associated with outcome variables.

All three models examining the relation between infant temperament (i.e., Negative Emotionality, Surgency, Regulation) and sleep quantity for 6-month-old infants with the covariate of in utero cortisol (AUCg) exposure were statistically significant, with AUCg posing as the only statistically significant predictor for each model (see Table 3 and Table 4). Specifically, greater maternal cortisol concentrations prenatally predicted less infant sleep duration at 6 months. Greater AUCg was also related to less frequent breastfeeding at 6 months. There were no significant main or interaction effects of infant temperament and breastfeeding on infant sleep quantity at 14 months.

#### 3.4.2. Hypothesis 2

There will be significant main effects of easier infant temperament and less frequent breastfeeding on greater infant sleep quality at 6 months of age while controlling for indicators of in utero cortisol exposure that are associated with outcome variables.

All three models examining the relation between infant temperament (i.e., Negative Emotionality, Surgency, Regulation), breastfeeding frequency, and sleep quality at 6 months were statistically significant (see Table 3 and Table 4). The model featuring Surgency as a predictor variable indicated a statistically significant relation between greater breastfeeding frequency and decreased 6-month sleep quality. Additionally, although the interaction between Surgency and breastfeeding frequency did not significantly predict 6-month infant sleep quality, the negative relation between breastfeeding frequency and 6-month sleep quality was statistically significant for infants with Surgency scores at 16th (4.38) and 50th (4.87) percentiles, but not at the 84th percentile (5.51; see Table 4) when examining conditional effects.

Similarly, the interaction between Regulation and breastfeeding frequency did not significantly predict 6-month infant sleep quality, but the negative relation between higher levels of breastfeeding frequency and poorer sleep quality was statistically significant for those in the 50th and 84th percentiles for Orienting/Regulation (i.e., scores of 5.23 and 5.63, respectively; see Table 4) when examining conditional effects.

Likewise, Negative Emotionality and breastfeeding frequency did not significantly predict 6-month infant sleep quality. However, the negative relation between higher levels of breastfeeding frequency and poorer sleep quality was statistically significant for those in the 50th percentile for Negative Emotionality when examining conditional effects (i.e., score of about 3.11; see Table 4). There were no other statistically significant main, interaction, or conditional effects for the remaining models.

#### 3.4.3. Hypothesis 3

There will be significant main and interaction effects of easier infant temperament and greater breastfeeding frequency on greater infant sleep quality at 14 months of age while controlling for indicators of in utero cortisol exposure that are associated with outcome variables.

The 14-month models examining the relation between Negative Emotionality and Surgency aspects of temperament, sleep quality, and breastfeeding frequency were statistically significant (see Table 4). Moreover, there was a main effect of temperament on sleep quality in the Surgency model. Specifically, greater Surgency at 6 months was associated with greater 14-month sleep quality. Although the interaction between Surgency and breastfeeding frequency did not significantly predict 14-month infant sleep quality, the negative relation between greater breastfeeding frequency and poorer sleep quality was statistically significant for those with Surgency at the 50th (4.96) and 84th (5.44) percentiles (See Table 4) when examining conditional effects. There were no other statistically significant main, interaction, or conditional effects that emerged within this model (see Table 3).

There were also no other main or interaction effects that emerged within the model of Negative Emotionality. The conditional effects of this model revealed a pattern in which higher levels of breastfeeding frequency were associated with lower scores on sleep quality only for those with scores on Negative Emotionality at the 50th (3.00) and 84th (3.70) percentiles. Additionally, there were no statistically significant main or interaction effects that emerged with the model examining the relation between breastfeeding frequency, infant Regulation/Orientation, and sleep quality (see Table 3).

## 4. Discussion

The current study sought to add to the current empirical literature by examining the main and interaction effects of infant temperament and breastfeeding on infant sleep duration and quality in early (6 months) and late (14 months) infancy given that complex bidirectional relations among these variables may change over this time. Additionally, when applicable, models considered confounding maternal cortisol concentrations that are transmitted via breastfeeding and lead to biological effects on the infant that counter some of the positive effects of breastfeeding on infant temperament and sleep [34].

### 4.1. Breastfeeding Frequency, Temperament, and Infant Sleep

Compared to recent national data from a systematic review [52], the current sample daily breastfeeding frequency was relatively low at 6 months (3.71 feedings/day compared to 5–8 feedings/day) and 14 months (1.33 feedings/day compared to 2–6 feedings/day), though this may be due to significant variability in the current study sample. In reference to data provided by the creators of the IBQ [39], the current sample scores were similar for Surgency (current sample 6-month *M* = 4.95; reported sample 6–9-month *M* = 4.53), Regulation (current sample 6-month *M* = 5.19; reported sample 6–9-month *M* = 4.61), and Negative Emotionality (current sample 6-month *M* = 3.08; reported sample 6–9-month *M* = 2.77), though all scale scores were higher in the current sample.

Regarding sleep quantity, maternal reports suggest that infants in the current sample did not obtain the recommended amount of daily sleep at 6 months (9.53 h compared to 12–15 h), though the 14-month mean (10.58 h) was close to the minimum recommended amount at this age (i.e., 11–14 h) [3]. This aligns with data from the 2022 National Survey of Children’s Health suggesting that about 34% of children between 4 months and 5 years sleep less than what is recommended based on their age [53].

Although no normative data exist for the sleep quality measure, sleep quality and quantity steadily increased over time, though this change was not statistically significant. Sleep quality may have increased based on the reduction in breastfeeding frequency over infancy. Likewise, even though daily recommended sleep duration decreases from 6 to 14 months, the increase in the current sample may be due to greater sleep consolidation secondary to reduced breastfeeding duration and/or biological maturation, which also underlies greater consistency in circadian rhythms [6,29,30].

### 4.2. Maternal Cortisol

Covariate analyses examining the relations between indicators of maternal diurnal salivary cortisol concentrations (CAR and AUCg) in late pregnancy and current study primary predictors and outcomes revealed only one statistically significant association between a greater AUCg and lower 6-month infant sleep quantity. This may be because AUCg serves as an index of chronic physiological stress, whereas CAR captures more acute reactivity that may not have as much of a lasting effect on infant circadian rhythms [48]. Additionally, the results of a recent, large longitudinal study suggest that psychosocial stress during pregnancy is more robustly associated with infant/child sleep duration than other forms of sleep problems (e.g., trouble sleeping alone, going to sleep, and waking regularly at night) [54], which may be because the latter are shaped more by environmental factors like caregiving and bedtime routines. Notably, the association between greater prenatal AUCg and shorter daily sleep duration at 6 months was still statistically significant in models considering infant temperament, breastfeeding frequency, and the interaction between the two. This is consistent with prior research supporting a robust link between maternal prenatal psychophysiological stress and long-term infant/child sleep outcomes, which are exacerbated by genetic risk markers for insomnia [54].

### 4.3. Main and Interaction Effects

No statistically significant interaction effects emerged in any of the current study moderation models. However, there were two significant main effects that emerged in the models investigating the infant Surgency scale, which indicates that there is a relation between the variables. In particular, a greater breastfeeding frequency at 6 months was associated with decreased 6-month sleep quality while considering infant Surgency at 6 months and the interaction between breastfeeding frequency and Surgency. This negative relation between greater breastfeeding frequency and poorer infant sleep quality at 6 months of age is consistent with our *a priori* hypothesis and empirical findings linking greater breastfeeding frequency in early infancy to more fragmented or less consolidated sleep [6,29,30].

Additionally, greater 6-month infant Surgency was associated with better sleep quality at 14 months while considering breastfeeding frequency at 14 months and the interaction between breastfeeding frequency and Surgency. This finding also aligns with our *a priori* hypothesis and extends the extant literature by replicating the positive associations between infant Surgency and sleep quality in a larger, longitudinal cohort [15], extending follow-up past 1 year [14,18], and including a broader assessment of temperament beyond sensory reactivity [14].

### 4.4. Conditional Effects on Sleep Quality

#### 4.4.1. Surgency

Although none of the interaction terms in any of the moderation models were statistically significant, there were a few patterns of findings that warrant additional consideration and may inform future research. Specifically, at 6 months, a statistically significant negative relation was found between breastfeeding frequency and sleep quality for those with Surgency scores at or below the mean. This finding does not indicate a statistically significant interaction and should be interpreted cautiously. However, it is consistent with the *a priori* hypothesis that, while breastfeeding negatively influences sleep consolidation in early infancy, the influence on overall sleep quality may be buffered by positive infant temperament. Previous studies have demonstrated that infant temperament is an endogenous factor that influences infant sleep outcomes in a transactional manner while considering important parenting factors [55].

At 14 months, the conditional effect of breastfeeding frequency on sleep quality was negative at the 50th and 84th percentiles of 6-month Surgency. While these results should be interpreted in light of non-significant interactions, it is theoretically possible that, as Surgency increases, the positive association between breastfeeding frequency and sleep quality becomes weaker. This is not in line with our original hypothesis, but these patterns of findings would imply that, for infants with greater Surgency, temperament-related processes may play a stronger role in shaping sleep quality in later infancy than breastfeeding frequency. The changes in these relations from 6 to 14 months of age may be due to the typical pattern of decreased breastfeeding frequency over this developmental period as well as a shift in infants’ circadian and other forms of regulation from more externally to internally mediated [56]. However, more studies are needed to test these moderation hypotheses since patterns are based on conditional effects and not statistically significant interaction effects.

#### 4.4.2. Negative Emotionality

Similar to Surgency, there was a statistically significant, negative relation between greater breastfeeding frequency and poorer 6-month sleep quality, but only for those in the 50th percentile for Negative Emotionality. These results warrant careful interpretation given the non-significant interaction term, but the pattern of findings implies that the relation between breastfeeding frequency and sleep quality possibly may be weaker for those with higher or lower Negative Emotionality. This could be because those with more Negative Emotionality may already experience more sleep disruptions [15] and those with less Negative Emotionality may be easier to soothe, are less reactive, and have more stable sleep that is less influenced by feeding context.

The negative relation between breastfeeding frequency and sleep quality at 14 months was statistically significant only for those at or above the mean in Negative Emotionality scores. While the interaction term was non-significant, this pattern of findings suggests that future studies may want to examine whether the link between greater breastfeeding frequency and sleep quality at 14 months is attenuated as Negative Emotionality increases. Similar to Surgency findings, this pattern is consistent with a shift toward increasingly endogenous control processes from early to late infancy, potentially amplifying the role of temperament in shaping sleep quality [56].

#### 4.4.3. Regulation/Orientation

The conditional effect results suggest that, at 6 months, more frequent breastfeeding was related to poorer sleep quality for those with Orienting/Regulation scores at or above the mean. This finding is not in line with the *a priori* hypothesis, but it could plausibly suggest that the sleep quality of infants who are better regulated is more influenced by breastfeeding frequency than their less-regulated counterparts, who may have existing disrupted sleep for other reasons (e.g., due to difficulties regulating arousal and maintaining consistent cycles of sleeping).

This pattern of conditional effects was opposite at 14 months, such that a statistically significant negative relation was found between breastfeeding frequency and sleep quality for those with Regulation/Orienting scores at or below the mean. This suggests that breastfeeding negatively influences sleep consolidation in 14-month-olds with less Regulation abilities.

### 4.5. Sleep Quantity Null Findings

Contrary to hypotheses, the current study did not replicate existing studies supporting associations between sleep quantity and breastfeeding frequency [21,30] or infant temperament [17]. However, recent findings from a large cohort study [57] suggest that breastfeeding behavior between 6 and 12 months postpartum is more related to sleep fragmentation than nighttime sleep duration or 24 h total sleep duration. Thus, while sleep timing or patterns may shift based on breastfeeding frequency, sleep duration may not be significantly influenced.

Likewise, temperament may not influence sleep need as much as it influences recovery from sleep disruptions, which was better captured by the sleep quality variable in the current study. Prior studies that found significant associations between infant temperament and sleep duration included small sample sizes and investigated temperament traits instead of the temperament dimensions utilized in the current study (*n* = 20) [17]. As opposed to higher-order dimensions, these traits assess the frequency of specific behaviors across situations, may be more state- or context-dependent, and may thus be more influenced by infants’/children’s sleep. Moreover, some studies examining temperament and sleep duration found significant associations by coding total sleep duration dichotomously based on a 25th percentile cutoff to define short sleep [58]. Therefore, it may be that the effects of temperament on sleep duration are not linear and may be best captured using a cutoff that captures meaningful biobehavioral differences in infancy. Similarly, it may be that temperament better differentiates those receiving sufficient versus insufficient sleep than in relation to a diverse range of daily sleep values above and below a mean value. Altogether, disparate findings in the current study versus other published work may be due to how infant temperament and sleep variables are conceptualized and measured, which supports the use of multi-method assessments of various infant temperament and sleep constructs in future studies.

### 4.6. Strengths, Limitations, and Future Directions

The current study utilized a longitudinal sample including prenatal, 6-month, and 14-month data, in addition to well-validated measures of infant temperament and commonly used assessments of breastfeeding frequency and infant sleep. However, several limitations to the study methodology could be improved upon in future studies.

Most notably, the unique combination of variables used in current study analyses also led to smaller sample sizes (*n* = 44–79) than anticipated given session attrition. Therefore, future studies should try to recruit and retain larger samples and/or utilize data analytic or imputation methods that minimize the effects of partial missing data. Relatedly, the current study sample was limited in sociodemographic diversity, which restricts the generalizability of the results and the degree to which the sociocultural context can be considered at important ecological system levels. There were additional limitations with respect to methodological choices noted in the present study.

Namely, the current study only obtained maternal salivary samples for cortisol assays in the third trimester. Given that there are sensitive periods of fetal growth, it may be helpful to capture specific windows of in utero cortisol exposure throughout pregnancy as well as cortisol concentrations in breastmilk to better understand how the timing of these exposures uniquely influences infant sleep outcomes. In addition, all primary study variables (i.e., infant temperament, breastfeeding frequency, sleep quality and duration) were derived from self-report measures that may include reporting bias and associated error (e.g., recall bias). Therefore, future studies should integrate direct behavioral assessments and physiological measures (e.g., actigraphy data). This may be particularly helpful given that sleep quality is more difficult for caregivers to assess reliably and accurately than sleep duration. It would also be helpful to assess all variables (i.e., maternal cortisol, infant temperament, breastfeeding, and infant sleep) at every timepoint and to specifically ask about breastfeeding frequency during infants’ nighttime sleep.

Furthermore, based on age-typical decreases in breastfeeding frequency from 6 to 14 months postpartum, the latter variable evidenced a positive skew. Although data transformations were utilized to help normalize the variable’s distribution to test current study hypotheses, future studies could examine other facets of breastfeeding that may lead to more normally distributed variables and capture novel relations with infant sleep outcomes, such as breastfeeding status/type, duration, amount, timing, delivery mode, responsiveness, or use as a soothing method/bedtime routine. Moreover, future studies may consider a more comprehensive examination of breastfeeding to better differentiate between breastfeeding purpose (e.g., nutrition versus soothing) and schedule (e.g., feeding on demand versus a feeding routine), which may also help to clarify interaction effects.

Specific to the current study, the Negative Affectivity scale on the IBQ-R SF demonstrated a relatively low internal consistency reliability, so additional studies are needed to replicate associated findings, and it may be helpful to use a longer form of the IBQ-R. Moreover, the timing between the prenatal session and 6- or 14-month outcomes may have led to other variables impacting relations between maternal cortisol and infant sleep (e.g., sleeping arrangements, consistency of sleep routines, and family structure and stress), which could be explored in future research.

## 5. Conclusions

Taken together, the findings lend support for the transactional model of sleep development, highlighting the salient, bidirectional, and dynamic interplay among caregiver (i.e., psychophysiological stress and breastfeeding behavior) and infant (i.e., Extraversion and Negative Affectivity) biopsychosocial factors that predict short-term and long-term infant sleep outcomes. Specifically, the current study results highlight the importance of managing maternal perinatal stress to prevent negative influences on mothers and infants. Additionally, based on hypotheses about processes underlying current study associations integrated from the prior theoretical and empirical literature, the promotion of positive dyadic engagement between mothers and infants during and outside of breastfeeding may result in better infant sleep outcomes. This is in line with studies supporting links between maternal sensitivity, including sensitivity to infants’ distress/non-distress, intrusiveness, and detachment and infant sleep problems. In addition, empirically supported interventions for infant sleep problems include cognitive–behavioral strategies to help manage consistency in healthy, adaptive sleep routines/organization. The study results support caregiver and infant risk/resiliency factors that could be incorporated into such treatments as parental psychoeducation situated within a broader sociocultural context.

## Figures and Tables

**Table 1:** Sample sociodemographic characteristics.

Characteristic	6M *n* (%)	14M *n* (%)
Race/ethnicity (not mutually exclusive)		
White	72 (79.12%)	41 (80.39%)
Latina	13 (14.29%)	8 (15.69%)
Black	2 (2.20%)	1 (1.96%)
Native Hawaiian and/or Other Pacific Islander	2 (2.20%)	1 (1.96%)
Asian	1 (1.10%)	0 (0%)
Native American	1 (1.10%)	0 (0%)
Highest degree of education		
Junior high school (7th–9th grade)	1 (1.27%)	0 (0%)
Partial high school (10th–12th grade)	1 (1.27%)	0 (0%)
High school degree (including GED)	11 (13.92%)	6 (13.64%)
Partial college (min 1 yr or other specialized or technical training)	23 (29.11%)	8 (18.18%)
Standard college or university degree	33 41.77%)	22 (50.00%)
Graduate training with degree	10 (12.66%)	8 (18.18%)
Income		
<$5000	1 (1.27%)	0 (0%)
$5000–9000	0 (0%)	0 (0%)
$10,000–19,000	11 (13.92%)	6 (13.64%)
$20,000–29,000	12 (15.19%)	7 (15.91%)
$30,000–39,000	10 (12.66%)	5 (11.36%)
$40,000–49,000	8 (10.13%)	5 (11.36%)
$50,000–74,999	26 (32.91%)	14 (31.82%)
$75,000–99,999	6 (7.59%)	3 (6.82%)
≥$100,000	5 (6.33%)	4 (9.09%)
Relationship status		
Single/Never Married	5 (6.33%)	2 (4.55%)
Married	69 (87.34%)	40 (90.91%)
Divorced	1 (1.27%)	1 (2.27%)
Engaged	1 (1.27%)	0 (0%)
Committed Relationship	3 (3.80%)	1 (2.27%)

Note.The sample size was different for each session, with *n* = 79 and *n* = 44 at the 6- and 14-month sessions, respectively. 6M = 6-month session and 14M = 14-month session.

**Table 2:** Descriptive statistics and correlation table for study variables.

Variable	*M*	*SD*
1. Surgency	4.95	0.55
2. Regulating/Orienting	5.19	0.47
3. Negative Emotionality	3.08	0.63
4. Sleep quality at 6M	3.75	0.83
5. Sleep quality at 14M	4.14	0.68
6. Sleep quantity at 6M	9.53	1.94
7. Sleep quantity at 14M	10.58	1.23
8. Breastfeeding frequency at 6M	3.71	3.98
9 Breastfeeding frequency at 14M	0.94	0.27

Note. 6M = 6-month session and 14M = 14-month session.

**Table 3:** Regressions of associations between temperament, breastfeeding frequency, and sleep.

Variable	*F*	*R* ^2^	*df*	*p*
Surgency				
Sleep Quality				
6M *	5.13	0.207	3	0.0032
14M *	3.98	0.206	3	0.013
Sleep Quantity				
6M *	3.08	0.278	4	0.030
14M	0.56	0.034	3	0.641
Regulation/Orientation				
Sleep Quality				
6M *	3.55	0.153	3	0.020
14M	2.28	0.129	3	0.092
Sleep Quantity				
6M *	3.00	0.273	4	0.033
14M	0.45	0.028	3	0.716
Negative Affectivity				
Sleep Quality				
6M *	4.72	0.193	3	0.005
14M *	3.11	0.169	3	0.036
Sleep Quantity				
6M *	2.98	0.272	4	0.034
14M	1.28	0.074	3	0.292

Note. This table depicts the relations between the three independent variables of temperament (i.e., Surgency, Regulation/Orientation and Negative Emotionality) with a moderator variable of breastfeeding frequency corresponding with each respective time point (6 and 14 months) and the dependent variables of sleep quality and quantity at different time points; * *p* < 0.05.

**Table 4:** Main effect and interaction results.

Predictor Variable			Main Effects				Conditional Effects (In Percentiles)		
	*F*	*R* ^2^	Temperament	Breastfeeding frequency	Interaction	Cortisol	16th	50th	84th
			*b/SE*	*b/SE*	*b/SE*	*b/SE*	*b/SE*	*b/SE*	*b/SE*
Surgency									
1. SQ_6M	5.13 *	0.21 *	−0.0045/0.03	−0.066/0.03 *	0.012/0.006	N/A	−0.015/0.004 *	−0.009/0.003 *	−0.002/0.005
2. SD_6M	3.08 *	0.28 *	−0.58/0.62	−0.25/0.51	0.06/0.10	−5.98/1/83 *	0.03/0.08	0.06/0.06	0.10/0.08
3. SQ_14M	3.98 *	0.21 *	0.56/0.21 *	0.38/0.39	−0.09/0.07	N/A	−0.02/0.06	−0.07/0.04 *	−0.12/0.05 *
Regulation									
1. SQ_6M	3.55 *	0.15 *	0.062/0.050	0.012/0.036	−0.004/0.007	N/A	−0.006/0.005	−0.009/0.004 *	−0.01/0.004 *
2. SD_6M	3.00 *	0.27 *	0.98/1.27	0.58/0.78	−0.10/0.15	−5.96/1.84 *	0.11/0.09	0.06/0.07	0.02/0.11
3. SQ_14M	2.28	1.29	0.09/0.22	−0.40/0.34	0.06/0.06	N/A	−0.12/0.06 *	−0.08/0.04 *	−0.05/0.05
Negative Emotionality									
1. SQ_6M	4.72 *	0.19 *	−0.05/0.03	−0.007/0.019	−0.0003/0.006	N/A	−0.008/0.005	−0.008/0.004 *	−0.008/0.005
2. SD_6M	2.98 *	0.27 *	−0.45/0.67	0.008/0.40	0.022/0.21	−5.88/1.88 *	0.06/0.10	0.08/0.07	0.09/0.10
3. SQ_14M	3.11 *	0.17 *	0.04/0.19	0.19/0.16	−0.09/0.05	N/A	-0.03/0.05	−0.09/0.04 *	−0.16/0.06 *

Note. Breastfeeding frequency is X; temperament (i.e., Surgency, Regulation, Negative Emotionality) is the moderator variable; sleep quality (SQ) and sleep duration (SD) at 6 months (6M) are the outcome variables. The variables of SQ_6M and SD_6M underwent data transformations to meet assumptions of normality. SQ_6M was given a cube root transformation and SD_6M was winsorized. *n* = 63 for 6-month sleep quality analyses, *n* = 37 for 6-month sleep quantity analyses, and *n* = 50 for 14-month sleep quality analyses; * *p* <0.05.

## Data Availability

The data presented in this study may be available on request from the corresponding author. The data are not publicly available due to specific ethical and privacy considerations.
